# Deciduous DPSCs Ameliorate MPTP-Mediated Neurotoxicity, Sensorimotor Coordination and Olfactory Function in Parkinsonian Mice

**DOI:** 10.3390/ijms20030568

**Published:** 2019-01-29

**Authors:** Christopher Simon, Quan Fu Gan, Premasangery Kathivaloo, Nur Afiqah Mohamad, Jagadeesh Dhamodharan, Arulmoli Krishnan, Bharathi Sengodan, Vasanth Raj Palanimuthu, Kasi Marimuthu, Heera Rajandas, Manickam Ravichandran, Sivachandran Parimannan

**Affiliations:** 1 Brain Research Institute, School of Medicine and Health Sciences, Monash University, Sunway Campus, Selangor 47500, Malaysia; 2 Anatomy Unit, Faculty of Medicine, AIMST University, Semeling, Bedong, Kedah 08100, Malaysia; qfganptana@outlook.com (Q.F.G.); jagadeesh@aimst.edu.my (J.D.); arulmoli@aimst.edu.my (A.K.); 3 Pre-Clinical Department, Faculty of Medicine and Health Science, UTAR, Sungai Long Campus, Selangor 43000, Malaysia; 4 Department of Biotechnology, Faculty of Applied Sciences, AIMST University, Semeling, Bedong, Kedah 08100, Malaysia; prema@meluhagroup.com (P.K.); afiqah@meluhagroup.com (N.A.M.); 5 Meluha Life Sciences Sdn Bhd, Lot 1G-2G Kompleks Lanai, Putrajaya 62250, Malaysia; 6 Pathology Unit, Faculty of Medicine, AIMST University, Semeling, Bedong, Kedah 08100, Malaysia; bharathi@aimst.edu.my; 7 School of Pharmacy, Queen’s University Belfast, Northern Ireland BT9 7BL, UK; V.Palanimuthu@qub.ac.uk; 8 China Queens College (CQC), China Medical University Joint College (off-campus), Shenyang 110122, China; 9 Centre of Excellence for Omics-Driven Computational Biodiscovery, AIMST University, Bedong, Kedah 08100, Malaysia; aquamuthu2k@gmail.com (K.M.); heraadaas@gmail.com (H.R.); ravichandran@aimst.edu.my (M.R.)

**Keywords:** Parkinson’s disease, dental pulp stem cells, MPTP, intranasal delivery, behavioural analysis, tyrosine hydroxylase

## Abstract

Parkinson’s disease (PD) is a neurodegenerative disorder defined by progressive deterioration of dopaminergic neurons in the substantia nigra pars compacta (SNpc). Dental pulp stem cells (DPSCs) have been proposed to replace the degenerated dopaminergic neurons due to its inherent neurogenic and regenerative potential. However, the effective delivery and homing of DPSCs within the lesioned brain has been one of the many obstacles faced in cell-based therapy of neurodegenerative disorders. We hypothesized that DPSCs, delivered intranasally, could circumvent these challenges. In the present study, we investigated the therapeutic efficacy of intranasally administered DPSCs in a 1-methyl-4-phenyl-1,2,3,6-tetrahydropyridine (MPTP)-induced mouse model of PD. Human deciduous DPSCs were cultured, pre-labelled with PKH 26, and intranasally delivered into PD mice following MPTP treatment. Behavioural analyses were performed to measure olfactory function and sensorimotor coordination, while tyrosine hydroxylase (TH) immunofluorescence was used to evaluate MPTP neurotoxicity in SNpc neurons. Upon intranasal delivery, degenerated TH-positive neurons were ameliorated, while deterioration in behavioural performances was significantly enhanced. Thus, the intranasal approach enriched cell delivery to the brain, optimizing its therapeutic potential through its efficacious delivery and protection against dopaminergic neuron degeneration.

## 1. Introduction

Parkinson’s disease (PD) is regarded as the most common degenerative disorder of the aging brain, following Alzheimer’s [1,2]. It is mainly characterized by tremors, bradykinesia, rigidity, and postural instability that results primarily from the extended loss of dopaminergic (DA) neurons in the substantia nigra (SN) [3]. Currently there is no cure for PD and most available treatments aim to reverse the dopamine deficiency and relieve its symptoms [4,5]. At present, stem-cell-based therapies are being investigated for their ability to reproduce functional dopaminergic neurons and replace degenerated neurons in the SN [4,6,7,8,9]. However, the ability of stem cells to produce functional neuronal cells and promote therapeutic efficacy in vivo needs to be demonstrated before they can be applied clinically [10,11,12]. Thus, obtaining a high-purity population of dopaminergic-like cells is critical for the development of stem-cell-based therapies for PD. Although mesenchymal stem cells (MSCs) have previously been shown to exert neuroprotection through the secretion of nerve growth factors, they rarely differentiate into functional neural cells. Additionally, due to the low incidence of adult neural stem cells (NSCs) and issues with harvesting, the utilization of other stem cell types with neural potential is required to achieve neuroregeneration [12,13,14,15,16]. Recently, DPSCs have been increasingly gaining prominence in the field of stem cell therapy for neurodegenerative diseases [8,17,18,19,20,21]. In fact, among all exploitable stem/progenitor cells, these cells have emerged as one of the best choices due to unique properties such as easy accessibility, clonogenicity, self-renewal and potency. Indeed, due to their neural crest origin, DPSCs displayed high plasticity and are particularly able to differentiate towards neural lineage. In vitro neural differentiation studies of rat and human DPSCs have previously demonstrated that these stem/precursor cell populations were able to differentiate into neurons based on cellular morphology and expression of early neuronal markers [4,19,20,22,23]. Above all, in vivo studies further revealed that rat DPSCs, when transplanted into an adult rodent brain, survived and expressed neuronal markers [10,17,18]. Thus, the recent development of clinically applicable populations of DPSCs has provided an avenue to overcome the failure of endogenous repair systems and substitute new cells into the lesioned brain [10,12]. Nevertheless, there are several existing obstacles concerning the utilization of DPSCs before translation into clinical application is made possible. One of the potential challenges that currently exist for stem cell therapy is the lack of safe and efficient cell delivery methods [24,25]. It has been reported that graft survival, sufficient enrichment of therapeutic cells in the brain, and the avoidance of stem cell distribution throughout peripheral organs are greatly influenced by the method of delivery [13,14,15]. At present, the routes used for stem cell delivery to the brain are either invasive or inefficacious due to the blood‒brain barrier (BBB). Numerous complications have been stated previously in successfully administrating these stem cells for therapeutic purposes [25]. Lately, however, a few studies have explored the nasal system as a novel stem cell delivery route to the brain where the intranasal delivery of stem cells were able to circumvent the blood‒brain barrier (BBB) and directly target the brain to treat PD [16,26,27]. Intranasally delivered MSCs have been shown to migrate through the cribriform plate and into brain tissue via olfactory and trigeminal pathways. Not only were the stem cells located in discrete regions of the brain but the delivery of MSCs appeared to have a therapeutic effect on PD animal models [16]. Currently, there are not many studies on stem cells in neurological diseases using intranasal delivery as the route of stem cell delivery to the brain. Although previous studies have demonstrated the underlying biology and cellular properties of DPSCs, the long-term survival and therapeutic impact of intranasally delivered DPSCs on PD experimental models has remained unexplored [6]. In this study, we examined the survival and differentiation of these cells upon intranasal administration into MPTP-induced mice models of PD. Our findings revealed that DPSCs delivered via intranasal application survived for a period of one month, differentiated into dopaminergic-like cells, and gradually enhanced the previously depleted dopaminergic activity of the nigrostriatal system following MPTP toxicity. Above all, intranasally administered DPSCs prominently ameliorated the MPTP-induced deficits observed in sensorimotor coordination and olfactory function of Parkinsonian mice.

## 2. Results

### 2.1. Basic Characterization of Isolated DPSCs

**Figure 1:**
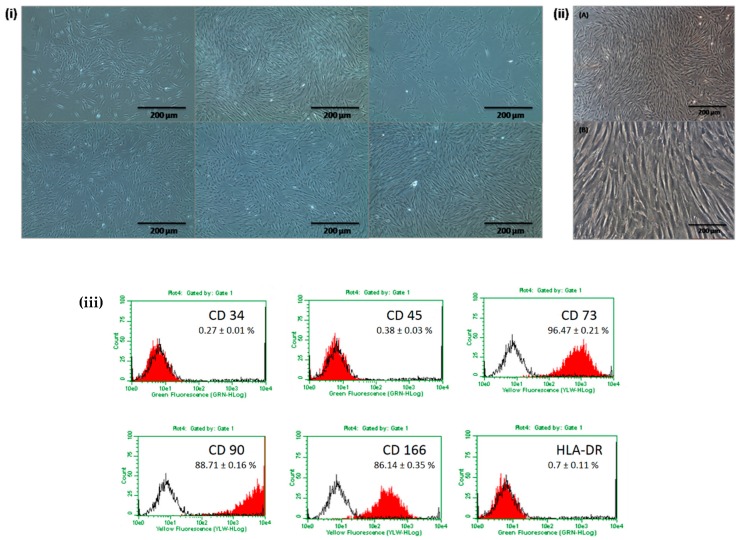
(**i**) Primary culture images obtained from 6 assays displaying the morphology of DPSCs (Magnification 4×; phase contrast images) (**ii**) Images of DPSCs expanded in FBS at subculture 3 (A: Magnification at 10×; B: Magnification at 20×; phase contrast images) (**iii**) Immunophenotype analysis of DPSCs expanded in FBS using flow cytometry. Cells were tested against human antigens CD34, CD45, CD73, CD90, CD166, and HLA-DR.

The isolated DPSCs displayed a fibroblastic-type morphology resembling to that of bone marrow MSCs (Figure 1i,ii). The cells formed a homogenous monolayer of adherent, spindle-shaped, fibroblast-like cells that proliferated fast and reached confluency after 10–12 days. Flow cytometry analysis further revealed that these cells expressed characteristic antigens of MSC-like cells including CD73, CD90 and CD166 but did not express HLA-DR and hematopoietic markers CD34 and CD45 (Figure 1iii).

### 2.2. Differentiation of DPSCs into Dopaminergic Neuron-Like Cells

**Figure 2:**
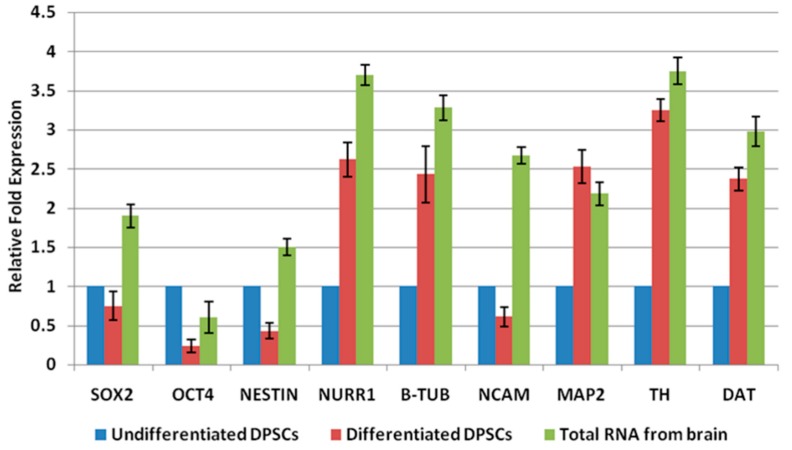
Detection of pluripotent indicators as well as neuronal markers. The Ct value of genes was analysed in the study using SYBR green-based qRT–PCR for DPSCs. Generally, the higher a fold change value, the more copies are present in the specific sample. Total RNA from brain was used as a positive control. Values are presented after normalization to 18s mRNA levels (*p* < 0.05).

Real-time PCR analysis indicated that gene expression profile of DPSCs was more consistent with mature neuronal cells, following neuronal induction. Pluripotent markers SOX 2, OCT-4 and early neuronal marker NESTIN were slightly downregulated, while mid-neuronal gene NURR1 and mature neuronal markers B-Tub, TH, DAT and MAP-2 had increased expression in induced DPSCs (Figure 2). Collectively, these data suggest that, in response to neuronal inductive stimuli, a greater proportion of DPSCs had stopped proliferating and had acquired a phenotype resembling mature neurons.

### 2.3. Recovery of Neurological Behaviour in Parkinsonian Mice Following Intranasal Application of DPSCs

**Figure 3:**
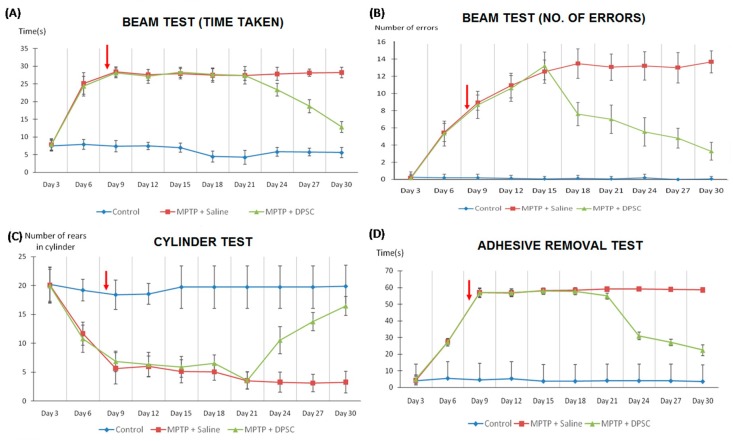
Control, MPTP-induced and DPSC-administered MPTP mice were tested for sensorimotor coordination on the challenging beam traversal, spontaneous activity in cylinder and adhesive removal tests. (**A**) The time taken to traverse the beam, (**B**) the number of errors made per step, (**C**) the number of spontaneous rears made on the hindlimbs and (**D**) the time taken to make contact with the sensory stimuli were measured. MPTP mice took longer to traverse the beam and made more errors during steps compared with the control mice in the beam test. In addition, MPTP mice were less active in the cylinder test and were significantly slower to respond to sensory stimuli compared with the control mice in the adhesive removal test. However, performance was significantly improved in MPTP mice across all measures following DPSC delivery (red arrow) at Day 7 (*p* < 0.001). Values are expressed as mean ± SD.

**Figure 4:**
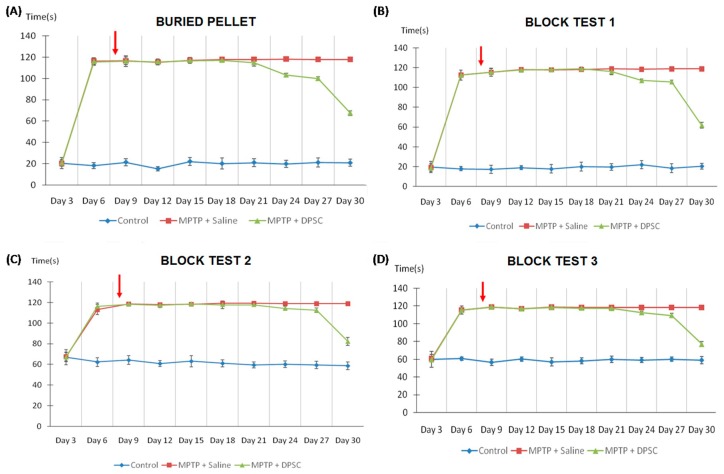
Control, MPTP-induced and DPSC-administered MPTP mice were tested for olfactory function on the buried pellet test and the block test. The time taken to (**A**) discover the hidden pellet and (**B**–**D**) to discriminate between their own scent and to that of a conspecific were measured. The block test was divided into three levels with increased complexity. MPTP mice took longer to find the hidden pellet and recognize foreign odour when compared with control mice in the buried pellet and block tests respectively. However, olfactory function was significantly improved in MPTP mice across both tests following DPSC delivery (red arrow) at Day 7 (*p* < 0.001). Values are expressed as mean ± SD.

MPTP impaired sensorimotor coordination in mice following its administration at day 0, as shown in the time taken to (A) traverse the beam, (B) the number of errors made per step, (C) the number of spontaneous rears made on hindlimbs and (D) the time taken to make contact with sensory stimuli (*p* < 0.001) (Figure 3). However, intranasal delivery of undifferentiated DPSCs at Day 7 gradually reversed this impairment one week later, across all measures (*p* < 0.001). Similarly, MPTP reduced olfactory function in mice following its administration at Day 0, as shown in the time taken to Figure 3A discover the hidden pellet and Figure 3B–D discriminate between their own scent and to that of a conspecific (*p* < 0.001) (Figure 4). The olfactory performance was significantly improved in MPTP mice across both tests following DPSC delivery at Day 7 (*p* < 0.001).

### 2.4. Intranasal DPSC Application Rescued Dopaminergic Neurons from MPTP Toxicity

**Figure 5:**
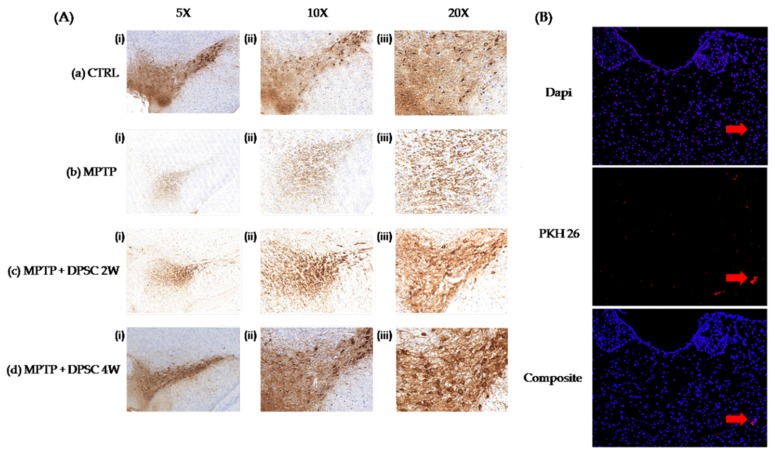
(**A**) Pictures displaying the effects of MPTP and intranasally administered DPSCs on tyrosine hydroxylase (TH) expression in the substantia nigra pars compacta (SNpc) of MPTP induced mice. (i–iii) Photomicrographs showing TH expression within the SNpc at low (5×), medium (10×) and high magnification (20×). (**a**): Control group at day 0 (**b**): Vehicle group: MPTP + saline at Day 7 (**c**) Treatment group: MPTP + DPSCs (two weeks after DPSC delivery) (**d**)Treatment group: MPTP + DPSCs (four weeks after DPSC delivery). The DPSCs labelled with PKH26 were delivered to MPTP mice at Day 7. Mice were lesioned with MPTP at Day 0. (**B**) Survival and migration of the PKH-labelled DPSCs within the SNpc following intranasal administration. PKH26 fluorescence visualization proved the existence of cell deposits in the SN (red arrow) of all grafted animals, indicating cell survival for at least four weeks after intranasal delivery (photomicrographs at high (20×) magnification).

The MPTP injection was targeted at the Substantia Nigra (SN) as illustrated in the coronal mesencephalon sections immunostained for TH displaying the varying degrees of degeneration in Figure 5A. MPTP exposure at Day 0 led to a marked loss of TH-positive neurons one week later within the SN for the vehicle group. In the control group, TH immunohistochemical staining was highly expressed in the cytoplasm of dopaminergic neurons, whose processes were elongated and stained clearly. In MPTP-treated animals, dopaminergic neurons in SN showed light sparse TH-immunostaining with short and disorderly processes. Following the treatment of undifferentiated DPSCs, there were gradual enhancements of TH immunoreactivity every week for four weeks from the time of DPSC administration. The mice treated with intranasal DPSCs also showed increased structural integrity and more condensation of immunoreactivity in the SN than that of the MPTP vehicle group.

### 2.5. Fluorescent Imaging of DPSCs In Vivo

PKH26-labelled DPSCs were intranasally administered at Day 7 and, even after four weeks, labelled cells were detected and primarily distributed within the SN (red arrow) (Figure 5B). This suggests that DPSCs showed the ability to migrate, engraft and survive within the SN.

## 3. Discussion

The in vitro differentiation ability of DPSCs towards dopaminergic-like cells and their neurorestorative capacities in MPTP-induced mice upon intrathecal administration has been shown previously [28,29]. In view of the difficulties associated with the intrathecal mode of delivery, the present study examined the neuroprotective efficacy of undifferentiated deciduous DPSCs in MPTP-induced PD mice following intranasal delivery. In a study by Danielyan et al. (2011), the beneficial effects of intranasal delivery of therapeutic MSCs were shown in PD animal models as a non-invasive alternative to the current traumatic surgical procedure of transplantation [16]. It was revealed that chronic treatment using the intranasal delivery method increased the number of delivered cells to the brain and enhanced its therapeutic benefit. Based on these results, we envisioned that the intranasal application of DPSCs might be more effective for developing therapeutic interventions for PD. Here, we demonstrated that the expression of cell surface and intracellular markers by DPSCs indicated their primitive features. The isolated stem cells from deciduous dental pulp tissue displayed a property of plastic adherence, expanded consistently and had a homogeneous fibroblastic morphology that was similar to that of bone marrow MSCs [4,30,31]. These stem cells shared a common mesenchymal marker profile with MSCs derived from bone marrow [18,29,32,33]. Following neural induction in vitro, the induced cells were characterized by an increase in mature neuronal markers, accompanied by a simultaneous decrease in early neuronal markers. Thus, our in vitro studies support the notion that at least a proportion of stem cells derived from dental pulp tissues may be capable of differentiating into neuronal cells when cultured under appropriate inductive conditions.

Remarkably, upon intranasal administration, the stem cells from dental origin effectively protected against the MPTP-induced deficits observed in behavioural assessments that examined sensorimotor coordination and olfactory function. In the first week following MPTP treatment, sensorimotor tests and olfactory assays displayed significant decreases in performance, which was a direct result from the loss of nigral dopaminergic neurons induced by MPTP neurotoxicity. This reduction however, was counteracted by the intranasal management of undifferentiated DPSCs on Day 7, as a marked progressive improvement was observed in sensorimotor coordination and olfactory function on mice treated with DPSCs, compared to the vehicle-treated animals. Additionally, we found that differentiated DPSCs protected against the loss of TH-positive neurons by migrating towards the SN and gradually attenuating the reduction in TH-positive neurons induced by MPTP neurotoxicity. Taken together, we have shown that DPSCs applied via the nasal route were able to migrate, survive, integrate into the host brain and appropriately differentiate into dopaminergic-like cells. Above all, the intranasal delivery of DPSCs enhanced cell delivery to the brain and optimized the therapeutic potential of DPSCs by protecting against dopaminergic neuronal degeneration and improving host neurological function. These findings, along with those of other studies concerning the migration and fate of undifferentiated DPSCs in vivo, demonstrate the great promise of this emerging paradigm. Nonetheless, before this potential can be realized in a clinical setting, future studies should include an in depth understanding of the molecular mechanisms governing their migratory properties and above all, the pattern and kinetics of DPSC colonization across various brain regions if these cells are to be used therapeutically in humans. The study on distribution of DPSCs across various brain regions would be particularly interesting taking into consideration that the presence of these cells is low in the mesencephalon. Our current study has not investigated the complete distribution of DPSCs in the brain, as it was beyond the scope of the study. The efficient migration and colonization of DPSCs toward regions of pathology in the brain followed by their successful integration, differentiation, and long-term survival at these sites is required for effective stem cell-mediated treatment of PD. Currently, there is a lack of consistency in certain areas of DPSC therapeutics and the potential of immunomodulatory properties of DPSCs is remarkable in order to form the basis of future therapeutics. Most studies conducted till date have not clearly displayed much support towards differentiation and engraftment but unanimously supported its immunomodulating properties. Thus far, it is evident that DPSCs are able to modulate immune cells and escape immune rejection, depending on its microenvironment. Recent reports have proposed that the inflammatory environment associated with PD might alter the DPSC polarization towards immunosuppressive or immunostimulating phenotype [10,20]. Consequently, further studies should be aimed towards understanding the mechanisms underlying immunomodulation by DPSCs to be able to utilize DPSCs for therapeutic purposes.

## 4. Materials and Methods

### 4.1. Isolation and Culture of DPSCs

The DPSCs were obtained from Hygieia Therapeutics Sdn. Bhd, Putrajaya, Malaysia and the following procedure was employed by them to isolate and culture DPSCs that were provided for our study. Upon receiving informed consent from donors’ parents, sound intact deciduous molars were extracted from children (5–8 years of age) undergoing planned serial extractions (*n* = 5) at the Department of Children Dentistry and Orthodontics, Faculty of Dentistry, University of Malaya. Samples were obtained under a protocol that was approved by the Medical Ethics Committee, Faculty of Dentistry, University of Malaya (Medical Ethics Clearance Number: DFCD0907/0042[L]; Date of approval: 01/09/2009). DPSC primary cultures from deciduous teeth were established as previously described by Govindasamy et al. (2010) [22]. In brief, cells were cultured in identical culture condition, with culture medium containing 1× KO-DMEM, 200 U/mL and 200 µg/mL of penicillin/streptomycin (Invitrogen, Carlsbad, CA, USA); 0.01× Glutamax (Invitrogen) and 10% foetal bovine serum (FBS) (Invitrogen) with humidified atmosphere of 95% of air and 5% of CO_2_ at 37 °C. Non-adherent cells were removed 48 h after initial plating. The medium was replaced every three days until the cells reached 80–90% confluency.

### 4.2. Flow Cytometric Analysis

Immunophenotyping of deciduous DPSCs were examined using flow cytometry at passage 5 [22]. The antibodies used to mark the cell surface epitopes were CD90-phycoerythrin (PE), CD73-PE, CD166-PE and CD34-PE, CD45-fluoroisothiocyanate (FITC), and HLA-DR-FITC (all from BD Pharmingen, San Jose, CA, USA). All analyses were standardized against negative control cells incubated with isotype-specific immunoglobulin (Ig) G1-PE and IgG1-FITC (BD Pharmingen). At least 10,000 events were acquired on a Guava Technologies flow cytometer, and the results were analysed using Cytosoft, Version 5.2 (Guava Technologies, Hayward, CA, USA).

### 4.3. Induction of Dopaminergic Neuronal Differentiation

DPSCs were subjected to neuronal induction using chemically defined media as described by Wang et al. (2010) [34]. Briefly, DPSCs were co-cultured with conditioned medium (CM) of ReNCell VM (Merck Milipore, Darmstadt, Hesse, Germany) for seven days before being exposed to Neuronal Media A containing Neurobasal A, B27 supplement, 20 ng mL^−1^ basic fibroblast growth factor (bFGF) and 20 ng mL^−1^ epidermal growth factor (EGF) for nine days, and Neuronal Media B containing Neurobasal A, 200 ng mL^−1^ sonic hedgehog (SHH), 100 ng mL^−1^ fibroblast growth factor 8 (FGF8), 10 ng mL^−1^ brain-derived neurotrophic factor (BDNF) and 10 µmol L^−1^ forskolin (Sigma-Aldrich, St. Louis, MO, USA) for seven days. All chemicals were purchased from Invitrogen unless stated otherwise.

### 4.4. Real-Time Polymerase Chain Reaction

Total RNA was extracted at the end of induction period using TRIzol (Invitrogen) and converted to cDNA according to Govindasamy et al. 2010 [22]. Gene expression levels were quantified in duplicates via real-time PCR, using SYBR Green Master Mix (Applied Biosystems, Foster City, CA, USA). PCR reactions were carried out on ABI 7900HT RT–PCR system (Applied Biosystems), and the results were analysed with the SDS v. 2.1 software. Gene expressions were analysed via comparative CT Method (ΔΔ*C*t) and were normalized to 18s rRNA. The gene expression was compared against undifferentiated cells as a control group and the primer sequences are listed in Table 1.

### 4.5. Animals

Sixty male Swiss albino mice (25–30 g and 10–12 weeks old) were randomly assigned into three groups: Control, MPTP-treated and MPTP-treated-intranasal DPSC. Mice were housed five per cage with food and water ad libitum under fixed temperature (25 ± 2 °C) and humidity (60 ± 5%) on a 12-h light/dark cycle. Ethical approval was obtained from the AIMST University Human and Animal Ethics Committee (AUHAEC) (application Ref. no.: AUHAEC6/FOM/2014; Date of approval: 19/06/2015).

### 4.6. Establishment of PD Model

The PD model was induced by injecting MPTP (20 mg/Kg; Sigma-Aldrich) in saline intraperitoneally, four times a day at 2-h intervals [35].

### 4.7. Intranasal Application of DPSCs

Intranasal application of DPSCs or vehicle (PBS) into MPTP-lesioned mice was performed seven days after MPTP injection. Prior to vehicle or cell treatment, mice were treated with 100 U of hyaluronidase (Sigma-Aldrich) dissolved in 24 µL sterile PBS as four repeated inoculations at 5-min intervals (3 µL in each nostril). One hour after pre-treatment with hyaluronidase, a DPSC suspension (5 × 10^5^ in 24 µL sterile PBS) or vehicle (PBS) was applied, following the same procedure [36].

### 4.8. Fluorescent Imaging of DPSCs in Vivo

DPSCs were tagged with PKH 26 (Sigma-Aldrich), as described by the manufacturer. In brief, cells were washed twice in a serum-free medium and mixed with a dye solution for 3 min. FBS was added for neutralization and suspended in saline. Tagged DPSCs were then introduced into mice via intranasal administration. Fluorescent images of harvested brain tissues were viewed under a fluorescence microscope (Olympus BX63 microscope; Olympus, Tokyo, Japan) to detect the presence of cells [9].

### 4.9. Behaviour Testing

All mice were pre-trained and two weeks prior to MPTP treatment, baseline recordings were obtained. Behavioural tests were carried out every three days following MPTP and DPSC treatment. Sensorimotor coordination was measured by the challenging beam traversal test, the spontaneous activity in cylinder test, and the adhesive removal test, as defined by Fleming et al. [37]. The challenging beam test was comprised of a beam with four sections that gradually decreased in diameter. Animals were trained to traverse the beam from the widest point to the narrowest and, during testing, a wire mesh grid was placed over the beam. Animals were videotaped while traversing the beam and the time taken to traverse the beam was determined. Spontaneous movement was measured by placing animals in a small transparent cylinder. The number of rear and hindlimb steps was measured. A rear was counted as when an animal made a vertical movement with both forelimbs removed from the ground, while hindlimb steps were counted when an animal moved both hindlimbs across the floor. As for the adhesive removal test, adhesive tape was applied on the snout of the animal and the time-to-contact and the time-to-removal were measured. To assess the odour detection ability and the olfactory memory, the buried pellet test and the block test were used [38]. The buried pellet test was conducted to examine whether the food-deprived animal was able to uncover the food pellet hidden beneath the cage bedding. The latency to uncover the buried food pellet beneath a layer of cage bedding, within a limited amount of time, was recorded. The block test, on the contrary, tested the ability of animals to discriminate between their own scent and that of a conspecific. The animal was presented with a wooden block scented with its own bedding and a block scented with another mouse’s bedding. The time spent in contact with each block was recorded. Slight modifications were made to the arrangement of the blocks in the block test to increase the level of difficulty of identifying the novel scent.

### 4.10. Perfusion and Fixation of Brains

At days 7, 14, 21 and 28 following intranasal application and MPTP treatment, mice were deeply anesthetized with ketamine/xylazine (Bioniche, Belleville, Toronto, ON, Canada) and perfused intracardially with saline (0.9%) followed by 4% paraformaldehyde (PFA). Brains were then collected, post-fixed at 4% PFA overnight, and transferred to 20% sucrose in 0.1 M phosphate-buffered saline (PBS) for cryoprotection. A set of coronal sections containing the SN (25 µm thickness) were cut on a microtome (Leica Microsystems, Wetzlar, Hesse, Germany) and stored at −20 °C.

### 4.11. Tyrosine Hydroxylase (TH) Immunostaining

The 25-µm coronal brain sections were rinsed twice in PBS, incubated in 0.2% Triton X-100 for 30 min at RT and rinsed three times with 0.5% bovine serum albumin (BSA) in 1× PBS for blocking. The brain sections were then incubated overnight at 4 °C with primary antibody: mouse anti-tyrosine hydroxylase (TH, 1:2000 dilution for brain tissue, Pel-freez, Rogers, AR, USA), rinsed three times in 0.5% BSA in 1× PBS and incubated with the appropriate biotinylated secondary antibody and avidinebiotin complex (Elite Kit; Vector Laboratories, Burlingame, CA, USA) for 1 h at RT. Bound antibodies were visualized by incubating with 0.05% diaminobenzidine-HCl and 0.003% hydrogen peroxide in 0.1M PB. Immunostained cells were analysed by bright-field microscopy [34].

### 4.12. Statistical Analysis

Results were presented as a comparison of average ± standard deviation (SD). Results obtained from behavioural data tested for normal distribution assumption and one-way Analysis of Variance (ANOVA) were used, followed by multiple post hoc comparisons among groups were made using Tukey’s or Bonferroni test. A *p*-value of <0.001 was considered significant. Data were analysed using SPSS version 22.0.

## 5. Conclusions

In conclusion, we have demonstrated that the intranasal delivery of DPSCs decreased brain lesion volume by promoting the formation of a ‘neurogenic niche’, ultimately leading to reconstruction of the Substantia nigra pars compacta. The intranasal approach enriched cell delivery to the brain, optimizing its therapeutic potential by protecting against dopaminergic neuronal degeneration, which is evident in the significant improvement of host neurological function.

## Figures and Tables

**Table 1:** List of genes with primer sequence and their product size.

Gene Name	Forward Sequence (5′–3′)	Reverse Sequence (5′–3′)	Base Pair Size
*SOX 2*	GGACAGTTACGCGCACATGA	AGCCGTTCATGTAGGTCTGC	188
*OCT 4*	TCCCGAATGGAAAGGGGAGA	GGCTGAATACCTTCCCAAATAGA	209
*NES*	GTAGCTCCCAGAGAGGGGAA	CTCTAGAGGGCCAGGGACTT	206
*NR4A2*	CGCCTGTAACTCGGCTGAA	AGTGTTGGTGAGGTCCATGC	169
*TUBB3*	GCGAGATGTACGAAGACGAC	TTTAGACACTGCTGGCTTCG	115
*NCAM*	TCTGCTAGCTCGTCTACCCC	AGCTTAGGTGCACTGGGTTC	110
*MAP2*	TAGAGGGTGTGATGGCTGAG	GGCAGAGGAAGGGATTTCTA	183
*TH*	TCATCACCTGGTCACCAAGTT	GGTCGCCGTGCCTGTACT	125
*DAT*	AAAGTCCTTTCCCGATGCGT	ATACCAGGACCCCCATCCTC	111
*18s rRNA*	CGGCTACCATCCAAGGAA	GCTGGAATTACCGCGGCT	186
